# Surgery methods and soft tissue extension are the potential risk factors of local recurrence in giant cell tumor of bone

**DOI:** 10.1186/s12957-016-0871-z

**Published:** 2016-04-19

**Authors:** Dongqi Li, Jinlei Zhang, Yi Li, Junfeng Xia, Yihao Yang, Mingyan Ren, Yedan Liao, Shunling Yu, Xiaojuan Li, Yan Shen, Ya Zhang, Zuozhang Yang

**Affiliations:** Bone and Soft Tissue Tumors Research Center of Yunnan Province, Department of Orthopaedics, The Third Affiliated Hospital of Kunming Medical University (Tumor Hospital of Yunnan Province), Kunming, Yunnan 650118 People’s Republic of China; Department of Orthopedics, Zhoukou City Central Hospital, The Affiliated Hospital of Xinxiang Medical College, Zhoukou, 466000 People’s Republic of China; Department of Oncology, Kunming General Hospital of Chengdu Military Command, Kunming, Yunnan 650118 People’s Republic of China

**Keywords:** Intralesional curettage, Extensive curettage, Local recurrence, Recurrence-free survival (RFS)

## Abstract

**Background:**

Various treatments of giant cell tumor of bone (GCTB) included in curettages and resections and with adjuvant are exerted, but the best treatment is controversial. The aim of the study was the identification of individual risk factors after various treatments in GCTB.

**Methods:**

A total of 179 patients treated for GCTB between 1998 and 2010 were concluded in the retrospective study. All patients were treated with intralesional curettage, extensive curettage, or wide resection. Mean follow-up was 60.2 ± 18.7 months (36~112 months). Age, gender, tumor location, Campanacci grade, soft tissue extension, pathological features, and surgical methods were performed to univariate Kaplan-Meier survival analysis and multivariate Cox regression analysis.

**Results:**

The local recurrence rates of intralesional curettage (41.9 %) and extensive curettage (19.0 %) were significantly higher than that of wide resection (7.7 %). The higher risk of local recurrence was found for soft tissue extension (hazard = 7.921, 95 % CI 1.107~56.671), compared with no statistical significances between gender, location, Campanacci grade, pathologic fracture, and local recurrences, which were shown by Kaplan-Meier analysis. However, recurrence-free survival (RFS) of patients younger than 30 was significantly lower than that of patients older than 30. The RFS of pathologic fracture patients with soft tissue extension was significantly lower than that of pathologic fracture patients without soft tissue extension. Multivariate Cox regression analysis indicated that the independent variable that contributed to recurrence-free survival was soft tissue extension and surgical methods. The RFS of extensive curettage had no statistically significant difference with wide resection and was significantly higher than that of intralesional curettage. Use of high-speed burring and bone cement significantly decreased the local recurrence rate.

**Conclusions:**

Age (below 30 years), gender, tumor location, Campanacci grade, and pathologic fracture have no statistically significant influence on local recurrences. Soft tissue extension and intralesional curettage of surgical methods increased the RFS. The results of the present study suggested that compared with curettage and wide section, treatment of GCTB by extensive curettage could provide the favorable local control and functional recovery.

**Electronic supplementary material:**

The online version of this article (doi:10.1186/s12957-016-0871-z) contains supplementary material, which is available to authorized users.

## Background

Giant cell tumor of bone (GCTB), or osteoclastoma, is a neoplasm with potential malignancy, accounting for approximately 5 % of all primary bone tumors and typically occurring in the epiphyses of long bones [1, 2]. Generally, GCTB consists of three cell types, mononuclear histiocytic cells, multinucleated giant cells, and neoplastic stromal cells [3], and has been classified into three grades by its histological appearances [4]. However, the clinical and prognostic value of the tumor’s grading has been disputed [5–8].

Although histopathological characteristics of most GCTB are benign, some types still have a high rate of local recurrence and the ability to metastasize with a recurrence rate of 2.5–45 % [9–12]. Those cases have postoperative recurrence within 24 months after the surgery [13, 14]. However, many studies used to show that X-ray grading, pathological fracture, and histological grading have no impact on tumor recurrence, invasiveness, and distant metastasis, which leads to the overlook and underestimation of the recurrence in practice. But with surgical methods being taken into consideration, more and more studies reveal that the recurrence rate of GCTB varies significantly with the factor [15–17] and debates on determining the best one retain for a long period.

It is reported that recurrence rates of GCTB would range from 0 to 65 %, depending on the type of treatment and local presentation of the tumor [17–19]. Generally, GCTB is always treated with intralesional curettage and wide resection. The former one has a low risk of invasiveness and can preserve the joint adjacent to the tumor [20], the recurrence rate of which is from 1 to 65 % [15–18, 21]. Other studies also report lower recurrence rate with the use of polymethylmethacrylate in intralesional curettage; however, the recurrence rate is similar to the result in the study of Blackley et al. and Turcotte et al. without using any adjuvant [16–20]. Wide resection is another recommended surgical therapy when the bone is extensively destructed or possibility to save the adjacent joint is small [22]. Lots of studies have suggested that wide resection contributes to the decrease in the risk of local recurrence as compared with intralesional curettage; moreover, wide resection may increase the recurrence-free survival rate to 84 to 100 % [17–19]. However, the wide resection is associated with higher rates of surgical complications and accompanied by considerable functional impairment.

In the present research, we retrospectively reviewed 179 GCTB patients treated with intralesional curettage, extensive curettage, and wide resection between 1998 and 2010. Recurrence rate was first determined according to different surgical methods. Log-rank test of Kaplan-Meier survival analysis was performed for clinicopathologic features and surgical methods. Multivariate Cox regression was used to analyze the risk factors of local recurrence and determine the best prognostic factors for recurrence.

## Methods

### Patients

We retrospectively identified 179 patients diagnosed as GCTB from 1998 to 2010 at the Third Affiliated Hospital of Kunming Medical University, including 99 male patients and 80 female patients. The average age of the patients was 32.0 ± 9.5 (13–64). The average follow-up time was 60.2 ± 18.7 months (36–112). The visiting intervals were 3 months for the first 2 years after surgeries, 6 months for the third to fifth years, and 12 months for patients after surviving the fifth year. Routine follow-ups included physical examination, X-ray examination, and chest computerized tomography (CT). The patients were not recalled specifically for the study; all the data were retrieved from medical records.

### Inclusion and exclusion criteria

Inclusion criteria covered the following: (1) Benign GCTB was confirmed by histopathological diagnosis according to the 2002 World Health Organization classification of GCTB [23]; (2) Patients had explicit imaging data including X-ray, CT, or magnetic resonance for diagnosis; (3) Patients had the complete records including diagnosis, therapy, follow-up, and recurrence. Exclusion criteria covered the following: (1) malignant GCTB; (2) the follow-up was shorter than 36 months.

### Classification of GCTBs

Levels of GCTBs were graded as grades I, II, and III according to the Campanacci method [15]. In our study, 25 patients were identified as grade I, 78 patients were identified as grade II, and 76 patients were identified as grade III.

### Surgery methods of GCTB patients

Patients enrolled in the present study were treated with intralesional curettage, extensive curettage, and wide resection. Intralesional curettages were performed as a wide cortical window was created to observe the tumor cavity and the tumor tissue was removed with a curette; for extensive curettage, chemical inactivation was performed on the basis of curettage and tumor cavity was packed carefully with autologous, allogenic bone grafts or polymethylmethacrylate (PMMA). According to Campanacci grade, soft tissue extension, and tumor location, the surgical methods of patients were determined. Forty-three patients were treated with curettage, 84 patients were treated with extensive curettage, and 52 patients were treated with wide resection.

### Data collection

Data were collected from the medical records and included in information on age, gender, tumor location, Campanacci grade, soft tissue extension, pathological fracture, and surgical methods (Table 3).

### Ethics

The project was approved by the Third Affiliated Hospital of Kunming Medical University. The ethics committee approved the relating screening, inspection, and data collection of the patients, and all subjects signed a written informed consent form. All works were undertaken following the provisions of the Declaration of Helsinki.

### Statistical analysis

Differences in the recurrence-free survival between different surgical methods or clinicopathologic features were calculated with log-rank test of Kaplan-Meier survival analysis. Multivariate Cox regression was used to analyze the risk factors of local tumor recurrence. Test of factor interactions was performed to identify potential confounding variables. Statistical analysis was performed using SPSS version 20.0 (IBM, Armonk, NY, USA).

## Results

### Local recurrence of patients treated with different surgical methods

Thirty-eight of 179 patients (21.2 %) had local recurrence of GCTB and the mean time to local recurrence was 16.9 ± 9.0 months (5–46 months) (as shown in Table 1). Six cases of recurrence patients were found with pulmonary metastasis (3.4%), 4 cases had local recurrence and pulmonary metastasis while 2 cases had pulmonary metastasis alone. As shown in Table 1, the patients treated with intralesional curettage had the highest recurrence rate, with 18 of 43 patients (41.9 %) found with local recurrence, while the recurrence rates of patients treated with extensive curettage and wide resection were only 19.0 and 7.7 %, respectively.

**Table 1:** Local recurrence rate treated with different surgical methods

Treatment	Total number	Recurrence	Recurrence rate (%)
Wide resection	52	4	7.7
Extension curettage	84	16	19.0
Abrasion + bone grafting	35	8	22.9
Abrasion + PMMA	49	8	16.3
Curettage	43	18	41.9
Alcohol	16	6	37.5
Iodine tincture or H_2_O_2_	27	12	44.4
Total	179	38	21.2

*H*_*2*_ *O*_*2*_ hydrogen peroxide, *PMMA* polymethylmethacrylate

### RFS analysis in patients by Kaplan-Meier

**Fig. 1:**
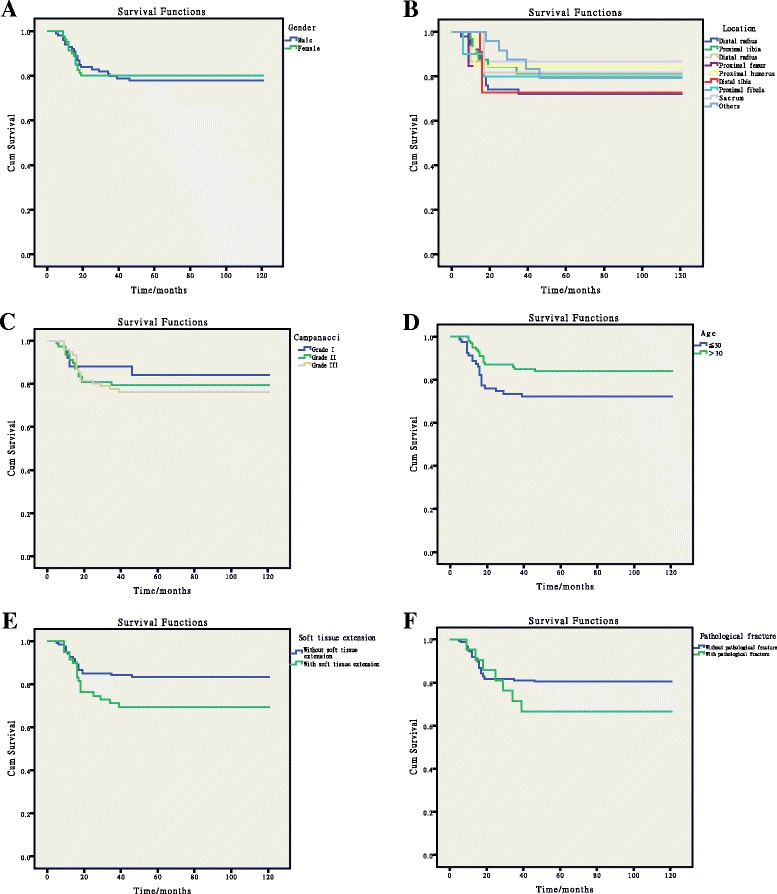
Results of Kaplan-Meier analysis and the log-rank test. **a** comparison between genders; **b** comparison among different locations; **c** comparison among different Campanacci grades; **d** comparison among different ages; **e** comparison between patients with soft tissue extension and patients without; **f** comparison between patients with pathological fracture and patients without

**Fig. 2:**
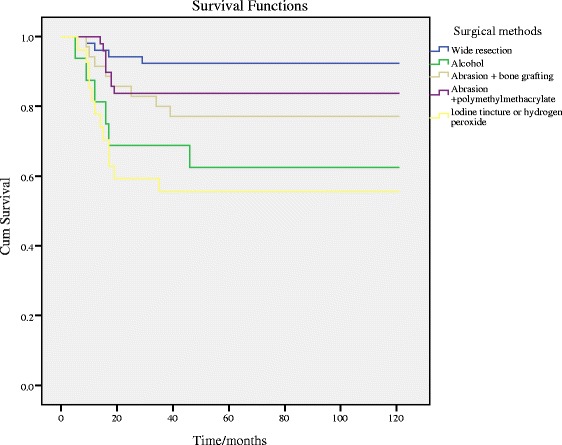
Kaplan-Meier analysis and the log-rank test among the influence of different surgical methods

There was no significant difference in RFS between different genders, tumor locations, Campanacci grades, or pathological fracture conditions (Fig. 1a–c, f). However, the RFS of patients below 30 years was significantly lower than that of patients older than 30 years (Fig. 1d). And the RFS of patients with soft tissue extension was significantly lower than that of patients without (Fig. 1e). Regarding surgical methods, a different therapy had dramatic difference impact on the RFS (Table 2; Additional file 1: Table S2; Fig. 2) and the pattern was similar to that of the recurrence rate.

**Table 2:** Recurrence-free estimates at 60 months based on different surgical methods

Treatment	Recurrence-free survival rate	SE
Wide resection	0.923	0.037
Extension curettage	0.810	0.043
Abrasion + bone grafting	0.771	0.071
Abrasion + PMMA	0.837	0.053
Curettage	0.581	0.075
Alcohol	0.625	0.121
Iodine tincture or H_2_O_2_	0.556	0.096
Total	0.788	0.031

*SE* standard error, *PMMA* polymethylmethacrylate, *H*_*2*_ *O*_*2*_ hydrogen peroxide

### Multivariate analysis by Cox regression

Our results showed that the gender, age, location, Campanacci grade, and pathological fracture had no influence on the recurrence rate. But soft tissue extension would increase the risk of having local recurrence (Table 3). Moreover, wide resection could significantly reduce the local recurrence risk in GCTB patients. And the patients treated with extensive curettage also got lower recurrence risk compared with those treated with curettage (Table 3).

**Table 3:** Hazard of recurrence in association with multiple factors

Parameter	Hazard	95 % CI	*P* value
Gender			
Male	0.921	0.469–1.807	0.810
Female	1.000		
Age			
≤30	1.595	0.800–3.178	0.185
>30	1.000		
Location			
Distal femur	2.242	0.630–7.791	0.212
Proximal tibia	0.659	0.129–3.361	0.616
Distal radius	2.049	0.284–14.766	0.477
Proximal femur	2.090	0.332–13.157	0.432
Proximal humerus	2.186	0.356–13.420	0.398
Distal tibia	2.736	0.593–12.867	0.195
Proximal fibula	3.325	0.501–22.073	0.213
Sacrum	0.411	0.058–2.903	0.373
Others	1.000		2.2
Campanacci grade			
I	2.199	0.234–20.672	0.491
II	3.000	0.405–22.196	0.282
III	1.000		
Soft tissue extension			
With extension	7.921	1.107–56.671	0.039
Without extension	1.000		
Pathological fracture			
With fracture	1.336	0.507–3.517	0.558
Without fracture	1.000		
Treatments			
Wide resection	0.044	0.011–0.175	0.000
Extension curettage			
Abrasion + bone grafting	0.144	0.044–0.471	0.001
Abrasion + PMMA	0.113	0.034–0.375	0.000
Curettage			
Alcohol	0.410	0.100–1.682	0.216
Iodine tincture or H_2_O_2_	1.000		

*PMMA* polymethylmethacrylate, *H*_*2*_ *O*_*2*_ hydrogen peroxide, *CI* confidence interval

## Discussion

GCTB is a severe type of tumor with a high recurrence rate, strong invasiveness, and complicated biological characteristic. Generally, it is quite difficult to predict the prognosis of the GCTB patients just with the methods of radiology, histology, or other clinical factors.

In the present study, the gender ratio was 1.24, including 99 male patients and 80 female patients, which was similar to the research of Niu [24]. This value varies significantly in different countries, from 0.8 in USA to 0.5 in Sweden [2]. However, we have found that gender was not a key factor influencing the recurrence-free survival rate of GCTB (Fig. 1a), while patients younger than 30 had a much lower recurrence-free survival rate than patients older than 30, which was indicative of the influence of age on the recurrence rate. This may be a result of the high level of bone metabolism of younger patients [25, 26]. But the result of Cox regression was opposite to Kaplan-Meier survival analysis and inferred that age was not an independent factor influencing the recurrence. When combined with other factors, it made no impact the recurrence rate of the patients factors (Table 3). The result of univariate Kaplan-Meier analysis was uncorrected on the account of possible confounding variates. To access the association of each variate with recurrence-free survival while controlling for the effects of other variates, multivariate Cox regression was performed. In our study, we found that age was not an independent factor of recurrence after Cox regression correction. Previous studies have also reported that recurrence rate is higher among patients with GCTB at distal radius [2, 18], whereas, according to our data, there was no significant difference among patients with different focus locations (Table 3). Moreover, another widely reported predictive indicator of GCTB, Campanacci grade [27], was also found to have no difference on the recurrence rate of GCTB in our study (Table 3). We thought this might result from the tendency of applying wide resection to high Campanacci grade patients in our study; 57.9 % of patients with grade III and 9 % of patients with grade II were treated with wide resection (Additional file 1: Table S1). We also reported that the pathological fracture conditions of patients had no influence on the recurrence rate (Additional file 1: Table S2). The only clinicopathologic factor contributing to the recurrence rate change was soft tissue extension. There were 25 patients with soft tissue extension in the present study, and the result of multivariate Cox regression is 7.021 (*p* = 0.039), indicating the increase of recurrence rate of GCTB due to soft tissue extension. It can be explained that performing the complete removal of tumor tissue is technically difficult and the current lack of applicable local adjuvants after surgery, which eventually leads to the high recurrence rate in GCTB patients with soft tissue extension [28, 29]; some alternative methods are imperative to solve this issue [30, 31].

Results of multivariate Cox regression showed that the surgical method was an independent factor influencing the recurrence rate. Among the three types of surgical methods, wide resection had the lowest recurrence rate (7.7 %) and the recurrence-free survival rate after 60 months was 92.3 %. However, restricted by some side effects, wide resection should not be taken as a standard method for GCTB treatment [32]. For curettage method, it has been concluded to have a high recurrence rate [14, 33], but our results were inconsistent with the previous studies. Patients treated with iodine tincture or hydrogen peroxide after the surgery had a recurrence rate of 44 %, and patients treated with alcohol had an even better prognosis (recurrence rate of 37.5 %). The difference in the recurrence rates between different adjuvants was not significant (Table 3). For the patients treated with extensive curettage, the two adjuvants, bone grafting and polymethylmethacrylate, both significantly reduce the recurrence rate compared with the curettage method. Previous studies attribute the good performance of extensive curettage to the application of abrasion and polymethylmethacrylate. In our study, the local recurrence rate of wide resection was significantly lower than that of intralesional curettage and extensive curettage (Table 1), and the recurrence-free survival rate of intralesional curettage was significantly lower than that of wide resection and extensive curettage (Additional file 1: Table S2). It indicated that wide resection and extensive curettage treatment were more applicable to patients than intralesional curettage based on lower recurrence rate and higher recurrence-free survival rate. However, wide resection treatment frequently causes serious complications including functional impairment of the extremities. There was no significance of recurrence-free survival rate between wide resection and extensive curettage (Additional file 1: Table S2). Taken together, extensive curettage should be a feasible and effective treatment for GCTB patients.

There were limitations in this study. Firstly, there was a lack of the detailed information of patients recalled or phone interviewed on account of the nature of retrospective data analysis. Secondly, the retrospective study was single center. Thus, the finding should be validated with a prospective, multi-center and larger size sample study, which aims to obtain the feasible and effective treatment for GCTB patients.

## Conclusions

In summary, age, gender, tumor location, Campanacci grade, and pathologic fracture had no statistically significant influence on local recurrences of GCTB. Soft tissue extension and intralesional curettage of surgical methods were independent risk factors of local recurrence of GCTB. The results of the present study suggested that compared with curettage and wide section, treatment of GCTB by extensive curettage could provide the favorable local control and functional recovery. Our study might provide potential guiding significance for the eligible treatment of GCTB in the future.

## Additional file

Additional file 1: Tables S1 and S2.
**Table S1.** Patient demographics. **Table S2.** Pairwise comparisons for the recurrence-free survival rate of different surgical methods. (DOC 65 kb)

## Abbreviations

CI: confidence interval
CT: computerized tomography
GCTB: giant cell tumor of bone
H_2_O_2_: hydrogen peroxide
PMMA: polymethylmethacrylate
RFS: recurrence-free survival
SE: standard error
