# Supplementary material for: Surgery methods and soft tissue extension are the potential risk factors of local recurrence in giant cell tumor of bone
Source: World J Surg Oncol. 2016 Apr 19;14:114. doi: 10.1186/s12957-016-0871-z (PMC4837597; doi:10.1186/s12957-016-0871-z)
Supplement: Additional file 1: Tables S1 and S2. — Table S1. Patient demographics. Table S2. Pairwise comparisons for the recurrence-free survival rate of different surgical methods. (DOC 65 kb) [file 12957_2016_871_MOESM1_ESM.doc]

**Additional file**

**Table S1.** **Patient demographics**

| **Parameter** | **Number** | **Percentage (%)** |
| --- | --- | --- |
| **Sex** |  |  |
| Male | 99 | 55.3 |
| Female | 80 | 44.7 |
| **Age** |  |  |
| ≤30 | 79 | 44.1 |
| >30 | 100 | 55.9 |
| **Location** |  |  |
| Distal femur | 50 | 27.9 |
| Proximal femur | 13 | 7.3 |
| Distal tibia | 11 | 6.1 |
| Proximal tibia | 32 | 17.9 |
| Proximal fibula | 10 | 5.6 |
| Distal humerus | 6 | 3.4 |
| Proximal humerus | 17 | 7.3 |
| Distal radius | 15 | 8.4 |
| Distal ulna | 4 | 2.2 |
| Proximal ulna | 5 | 2.8 |
| Phalanx | 4 | 2.2 |
| Scapula | 1 | 0.6 |
| Sacrum | 11 | 6.1 |
| Innominate bone | 4 | 2.2 |
| **Campanacci Grade** |  |  |
| Grade I | 25 | 14.0 |
| Grade II | 78 | 43.6 |
| Grade III | 76 | 42.5 |
| **Soft tissue extension** | 59 | 33.0 |
| **Pathological fracture** | 21 | 11.7 |
| **Treatments** |  |  |
| Wide resection | 52 | 29.1 |
| Extension curettage |  |  |
| Abrasion+bone grafting | 35 | 19.6 |
| Abrasion+PMMA | 49 | 27.4 |
| Intralesional Curettage |  |  |
| Alcohol | 16 | 8.9 |
| Iodine tincture or H_2_O_2_ | 27 | 15.1 |
| **Total** | 179 | 100 |

**Table S2. Pairwise Comparisons for the Recurrence-Free Survival Rate of Different Surgical** Methods

| **Treatment** | **Wide resection** | | **Extensive**  **Curettage** | | **Intralesional**  **Curettage** | |
| --- | --- | --- | --- | --- | --- | --- |
|  | χ^2^ | *P* | χ^2^ | *P* | χ^2^ | *P* |
| **Wide resection** | - | - | 3.146 | 0.076 | 15.895 | 0.000 |
| **Extensive Curettage** |  |  |  |  | 8.949 | 0.003 |
| **Intralesional Curettage** |  |  |  |  | - | - |
